# Dataset of lithium-ion cell degradation under randomized current profiles for NMC, NCA, and LFP chemistries

**DOI:** 10.1016/j.dib.2025.111531

**Published:** 2025-04-04

**Authors:** Mohsen Heydarzadeh, Teemu Toivola, Victor Vega-Garita, Eero Immonen

**Affiliations:** Turku University of Applied Sciences, Joukahaisenkatu 3, 20520 Turku, Finland

**Keywords:** Cell degradation, Lithium–nickel–manganese–cobalt-oxides (NMC), Lithium–nickel–cobalt–aluminium-oxides (NCA), Lithium–iron–phosphate (LFP)

## Abstract

This paper describes an experimental dataset of lithium-ion cells subjected to a randomized usage profile and periodically characterized through diagnostic tests. The study involved testing eight cells from three types of chemistries (NMC, NCA, LFP) over more than 600 full charge–discharge cycles. The dataset captures the cell's degradation process, including capacity fade and power loss. The experimental procedure comprised an initial full charge/discharge cycle to activate the cells, followed by repeated cycles at varying discharging current rates ranging from 0.5C to 2C/5C. Periodic Hybrid Dynamic Pulse Power Characterization (HPPC) and Constant Current∼ (CC) discharging profiles were executed as reference performance testing (RPT) to monitor transient dynamics and overall performance changes. Furthermore, the Constant Current–Constant Voltage (CC–CV) charging protocol was implemented at 1C charging rates. The experiment entailed the collection of data on voltage, current, cell temperature, ambient temperature, and time, with a 1 Hz sampling rate, utilizing specialized equipment, Chroma 1107 system and temperature sensors. The dataset facilitates the characterization of cell aging under various usage patterns, thereby enabling the development of models and management strategies for different applications. The data was collected at the New Energy Research Center at Turku University of Applied Sciences (TUAS), Finland.

Specifications Table

**Table:** 

Subject	Engineering & Materials science
Specific subject area	*Three types of lithium-ion cell degradation using a RC operation profile.*
Type of data	Table. Raw and processed.
Data collection	*The Chroma battery test supplies the user-defined current profile to the cell and records the output voltage and temperature. A cycle is defined by the following Steps 1 to 4:* *1) CC-CV charge at a constant 1C.* *2) CC discharge at 1C.* *3) HPPC discharge at 0.5C, 1C, and 2C.* *4) User-defined (RC) discharge profile to reach the cut-off voltage.*
Data source location	*New Energy Research Center, Turku University of Applied Sciences.* *City: Turku.* *Country: Finland.*
Data accessibility	Repository name: *FairData.* Data identification number: 10.23729/b07462e8-9e31-428c-bdd5-deb1890c608b Direct URL to data: Dataset - etsin.fairdata.fi
Related research article	*None.*

## 1 Value of the Data

• The experimental data involves collecting randomized current profiles, referred to as randomized cycles (RC), and diagnostic tests for three different types of cells including four ICR18650-26J (NCA), two INR21700-40T (NMC), and two JGPFR26650 (LFP) which are tested at room temperature.
• The RC aging experiments are designed to simulate various usage patterns that bring the battery State of Charge (SOC) from 100 % to 0 %.
• Diagnostic test, in the form of capacity tests, RPT and HPPCs are performed periodically to evaluate the cells' degradation.
• This dataset contains high-frequency measurements (at the 1 Hz frequency) of eight cells which were aged using a random current profile.
• These measurements were recorded aging process both during RC usage profiles and diagnostic cycles, giving insight and access to the performance-degradation behavior of the cells.

## 2 Background

Batteries technology and their role in the modern energy storage systems attract significant attention from researchers to study battery dynamics and the effects of various factors on their performance. The diverse chemistries of battery cells, each with unique characteristics, result in varied performance under different usage conditions [1]. Precise and reliable battery models enable battery management systems (BMS) to monitor and control batteries more efficiently and safely. Achieving this requires comprehensive data on battery performance under different conditions and an understanding of the cell's chemistry, which are indispensable for such studies.

## 3 Data Description

**Fig. 1:**
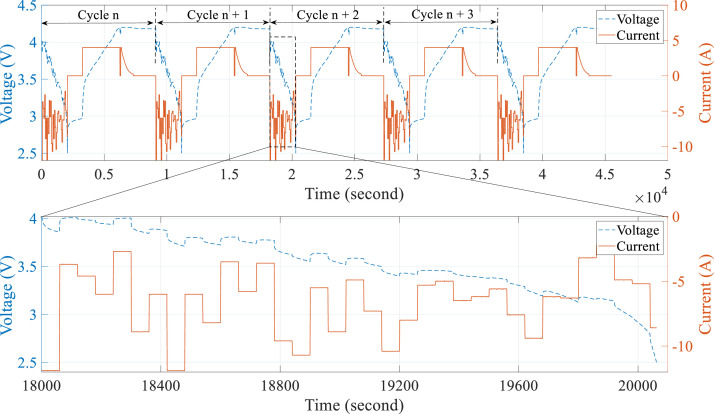
The battery cell current and voltage during the RC profile used to dynamically age the cell NMC_CELL_1 (where a positive current denotes battery charging, a negative current denotes battery discharging, while zero current denotes a resting).

The dataset comprises various usage profiles for eight cells, including INR21700-40T (NMC), ICR18650-26J (NCA), and JGPFR26650 (LFP) cells over more than 600 cycles. Detailed technical specifications for the cells and their respective quantities are provided in Table 1. To simulate the aging process experienced by the lithium-ion cells, the predominant charging/discharging profiles outlined in Table 2 were employed. The RC usage profiles consist of a series of discharge current values, with each of them applied for one minute. These values were pre-generated in MATLAB using a random function based on a uniform distribution, tailored to the specifications of each cell's types. The profiles were designed to replicate realistic operating conditions, ensuring that the cells aged under scenarios that closely resemble actual usage. Fig. 1 present the part of the RC cycling for NMC cell during five cycles. All cells are tested at room temperature.

**Table 1:** Technical specification of cells [[2], [3], [4]].

Cell Type	NMC	NCA	LFP
Manufacturer	SAMSUNG	SAMSUNG	JGNE
Model	INR21700-40T	ICR18650-26J	JGPFR26650
Size (diameter × length)	21.2 × 70.3 $mm^{2}$	18.4 × 65.0 $mm^{2}$	26.2 × 65.7 $mm^{2}$
Weight	70 g	45 g	83.5 g
Nominal capacity (Q_nom)	4.0 Ah	2.55 Ah	3.0 Ah
Nominal voltage	3.6 V	3.63 V	3.2 V
Charge cutoff-voltage	4.2 V	4.2 V	3.6 V
Discharge cutoff-voltage	2.50 V	2.75 V	2.50 V
Cutoff-current	200 mA	130 mA	150 mA
Quantity of cells	2	4	2

**Table 2:** Description of the main experimental cycle.

Cycle type	Action	Exit condition
1	CC–CV charging at 1C	Charging cutoff-volt/current reached
2	HPPC	Discharging cutoff-voltage reached
3	Ref. CC discharging at 1C	Discharging cutoff-voltage reached
4	RC usage profile	Discharging cutoff-voltage reached

Procedure I depicts the step by step of the experiment instruction. Activation of the new cells involved conducting two full cycles at a 0.5C. Furthermore, the batteries are subjected to low-current discharges (0.04A) to precisely monitor their open-circuit voltage∼ (OCV) as a function of the SOC, providing insights into their baseline behavior. The main body of the experiment involves subjecting the batteries to continuous discharge profiles at varying current rates (0.5C to 5C, and up to 2C for NCA cells), simulating various operational loads. Between each discharge, the batteries are recharged at a constant rate of 1C. Additionally, every 200 cycles, the RC data is updated to capture the evolving performance characteristics. After every set of 20 cycles, HPPC tests are performed at different current rates (0.5C, 1C, and 2C). This test involves a series of pulsed discharges at constant current rate for 6 min each, followed by rest periods, allowing to track changes in the battery's transient dynamics over time. Following the HPPC tests, reference constant current discharges at 1C is served as benchmarks for evaluating battery degradation. This procedure ensures a comprehensive analysis of the battery's behavior under different loads and over extended use, with regular checks to monitor how aging affects performance, such as capacity fade and power loss.

**Procedure 1:** Cell's degradation experiment procedure.

**Fig. 2:**
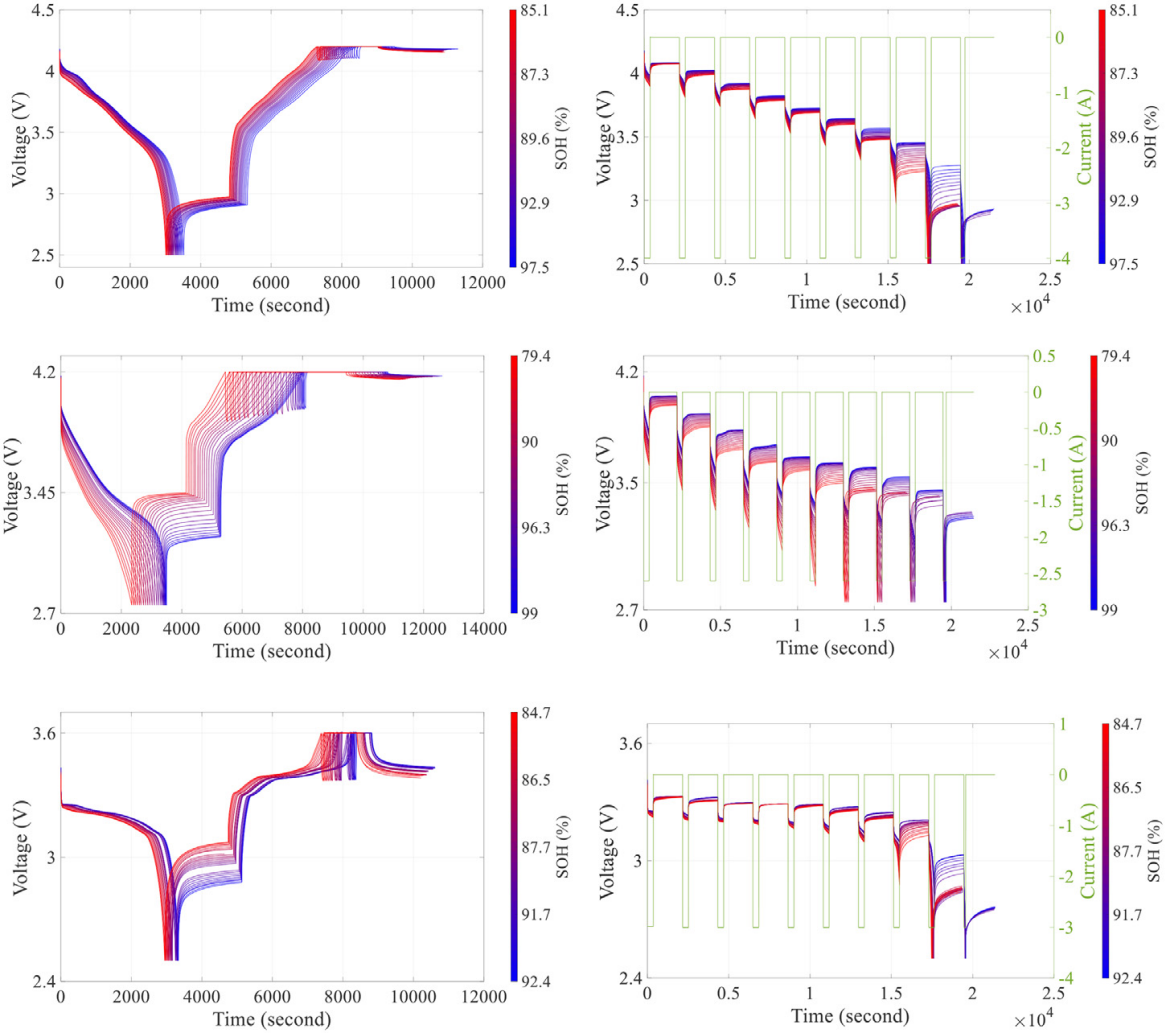
Comparison of RPT cycles at 1C for three different cell chemistries: NMC, NCA, and LFP. The first column shows the reference tests, while the second column illustrates the HPPC tests for each cell type. First row's figures represent results for NMC_CELL_1, second row for NCA_CELL_1, and third row for LFP_CELL_2.

Fig. 2 presents the aging effects on the cell's behavior in different types of the cells under the RPTs, while the color bar in the right side of each graph shows the cell's State of Health (SOH). For each cell, SOH is calculated from the capacity tests performed at each reference test. The discharged capacity, measured in Ah, and normalized with respect to nominal capacity defined as in Table 1, (Q_nom), is computed integrating the current I(t) with respect to time [5]:

$$SOH\;=\frac{1}{3600\;\times \;Q_{\text{nom}}\;}\;\int I\;\left(t\right)\;\;dt\;\times \;100\;\;\left(\%\right)$$

**Fig. 3:**
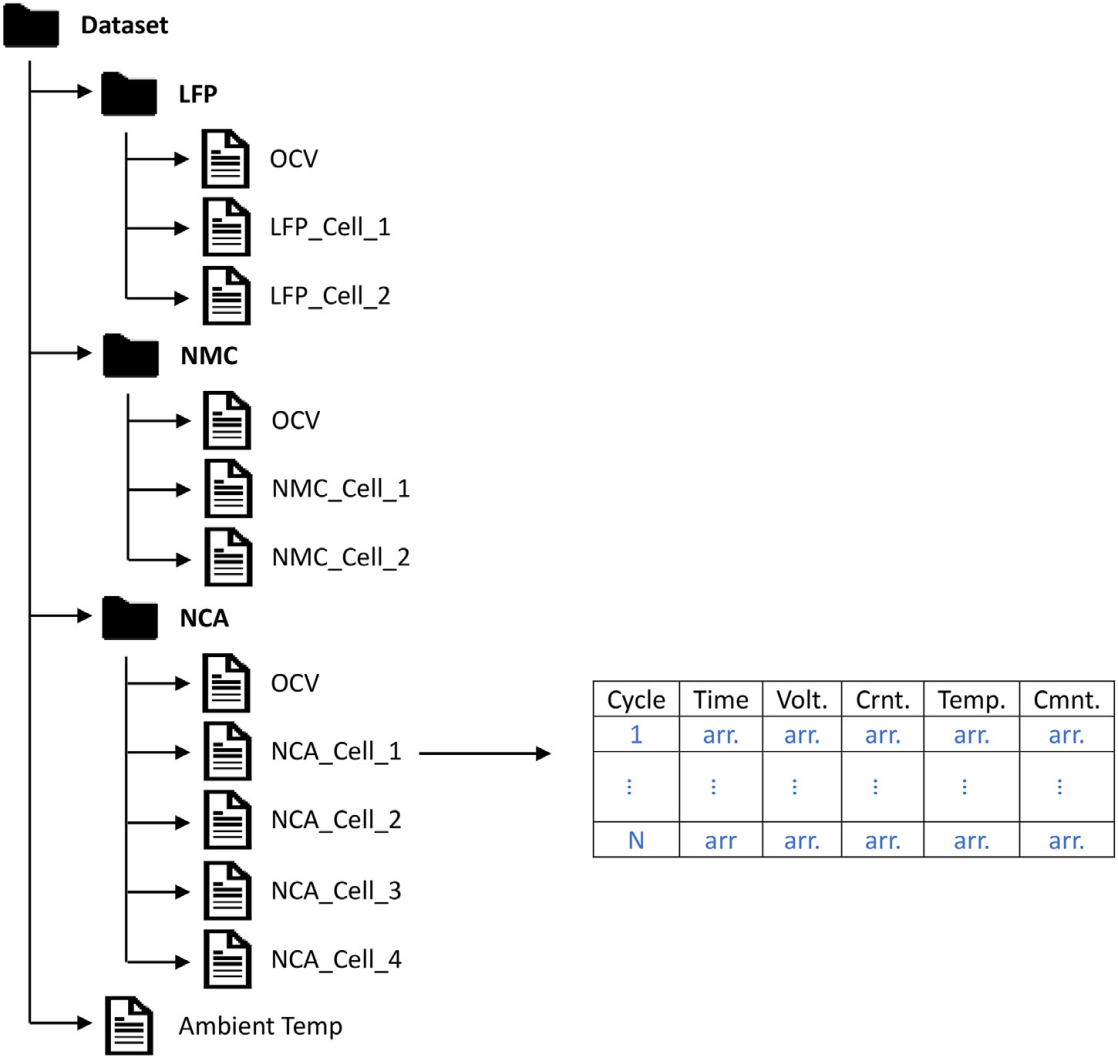
Dataset folder structure.

The dataset provides both raw (.xlsx) and processed (.mat) data. Each cell's raw data are saved in three excel files with two or three spreadsheets, that can be used to extract raw RPT and RC tests. Each excel file represents the unique RC discharging profile. The main limitation of using the raw data is the large size, that prevents fast data analysis and processing. To allow for fast data analysis, recorded signals are extracted from raw data and saved in .mat files. The general structure of the processed dataset can be seen in Fig. 3. It contains three main folders (one for each cell's chemistry type), each folder contains sub-folders for every cells were subjected to during the experiment. Those are, the folders ‘Cell1’, Cell2’, and ‘OCV’ contain full experiment's cycles of each cell and open circuit voltage of cells at low current discharging, respectively.

## 4 Experimental Design, Materials and Methods

**Fig. 4:**
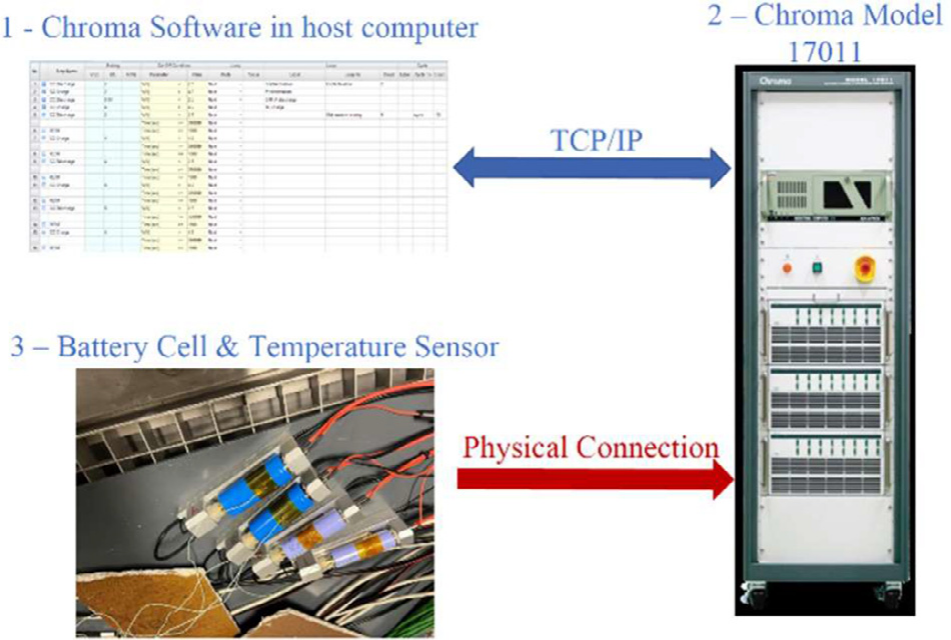
Battery degradation experiment set-up in New Energy Research Center.

RC cycles and RPT experiments are performed with the equipment available at the New Energy Research Center. Fig. 4 presents the schematic of the experiment setup, including the battery cell test system, Chroma 17011. Both cycling and diagnostic tests are designed via the Chorma software, which allows to define test instructions, i.e., the sequence of steps to be followed in order to perform an experiment. Each cell was tested inside the clamp and instrumented with a T-type thermocouple to measure the surface temperature in the center location.

## Limitations

There are two limitations of the dataset. First, in some cells, one part of the RPT cycle was lost due to issues with the experimental set-up. Second, the first RPT cycle was not performed exactly after the 20 RC cycles.

## Ethics Statement

The authors have read and followed the ethical requirements for the publication of Data in Brief, and hereby confirm that the work did not involve data collected from human subjects, animal experiments, or social media platforms.

## CRediT authorship contribution statement

**Mohsen Heydarzadeh:** Conceptualization, Data curation, Visualization, Writing – original draft, Formal analysis. **Teemu Toivola:** Conceptualization, Investigation, Data curation. **Victor Vega-Garita:** Conceptualization, Writing – review & editing. **Eero Immonen:** Supervision, Writing – review & editing.

## Data Availability

FairdataNMC, NCA, and LFP Cells Degradation Under Randomized Current Profiles (Original data)
